# Supplementary material for: Elaeocarpus sylvestris var. ellipticus Extract and Its Major Component, Geraniin, Inhibit Herpes Simplex Virus-1 Replication
Source: Plants (Basel). 2024 May 22;13(11):1437. doi: 10.3390/plants13111437 (PMC11174555; doi:10.3390/plants13111437)
Supplement: Supplementary file 1 [file plants-13-01437-s001.zip › plants-2917152-supplementary.pdf]

Herpes Simplex Virus-1 Replication Inhibitory Activity of *Elaeocarpus sylvestris* var. *ellipticus* extract and Its Major Component, Geraniin

Yeong-Geun Lee [1,†], Dae Won Park [2,†], Jeong Eun Kwon [1], Hyunggun Kim [3,*] and Se Chan Kang [1,*]

[1] Department of Oriental Medicine and Biotechnology, Kyung Hee University, Yongin 17104, Republic of Korea

[2] GENENCELL Co. Ltd., Yongin 16950, Republic of Korea

[3] Department of Biomechatronic Engineering, Sungkyunkwan University, Suwon 16419, Republic of Korea

[†] These authors contributed equally to this work (Y-G.L.; D.W.P.).

* Correspondence: hkim.bme@skku.edu (H.K.), sckang@khu.ac.kr (S.C.K.).

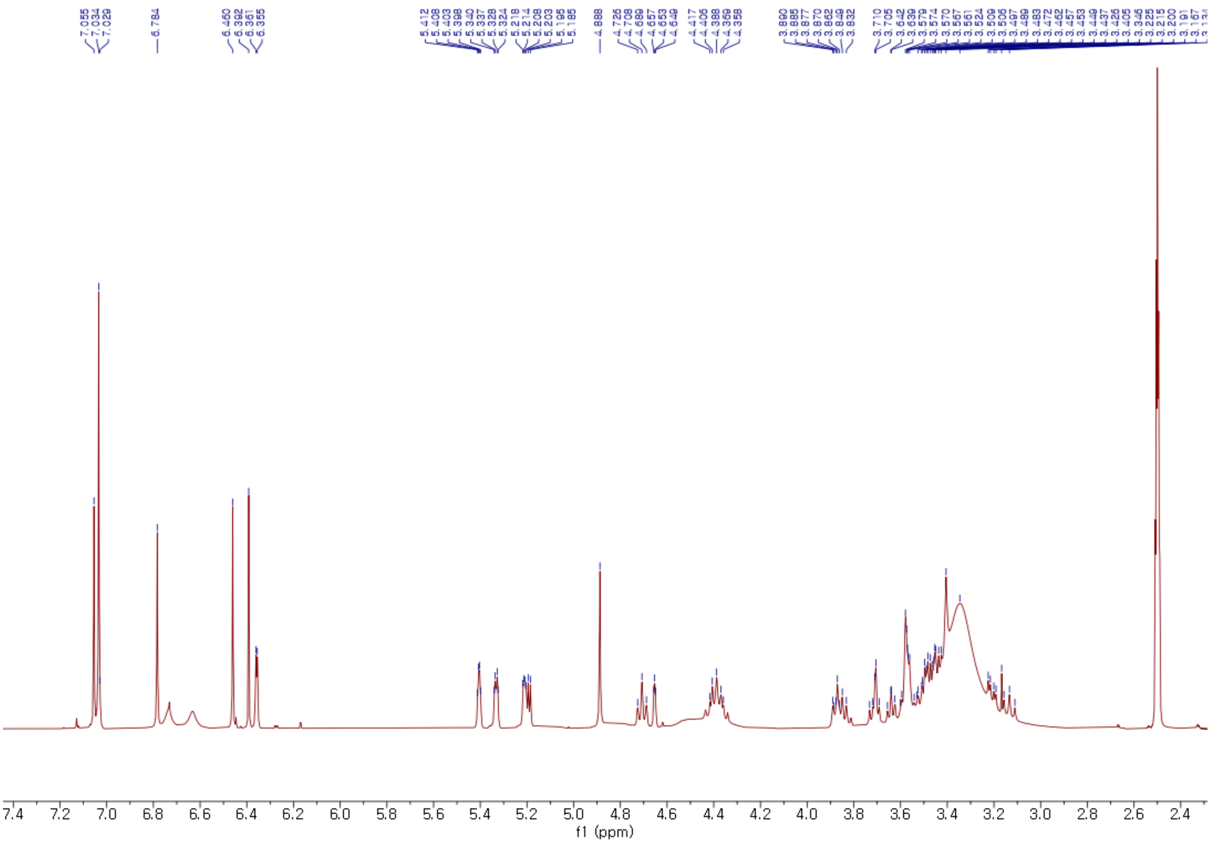

Figure S1. ^{1}H-NMR spectrum of geraniin (CD_3OD, 600 MHz).

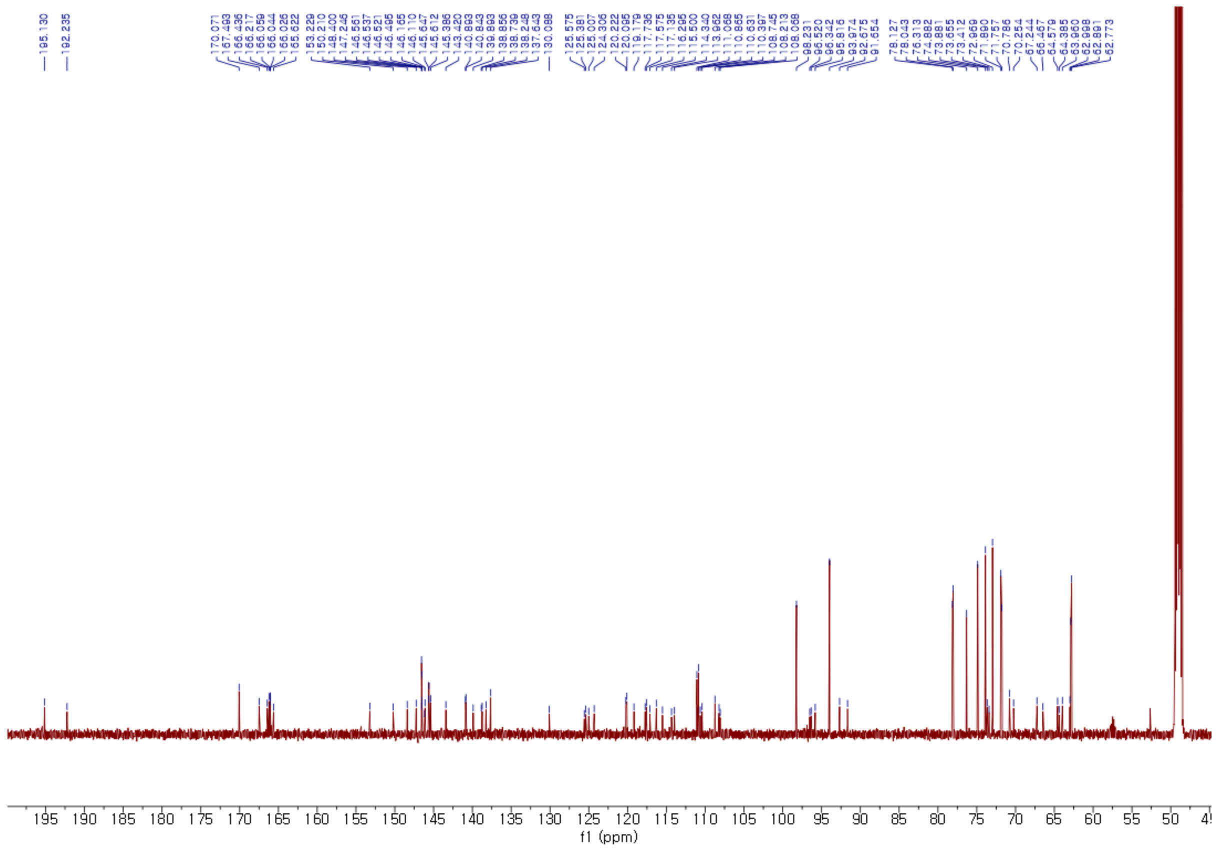

Figure S2. ^{13}C-NMR spectrum of geraniin (CD_3OD, 150 MHz).

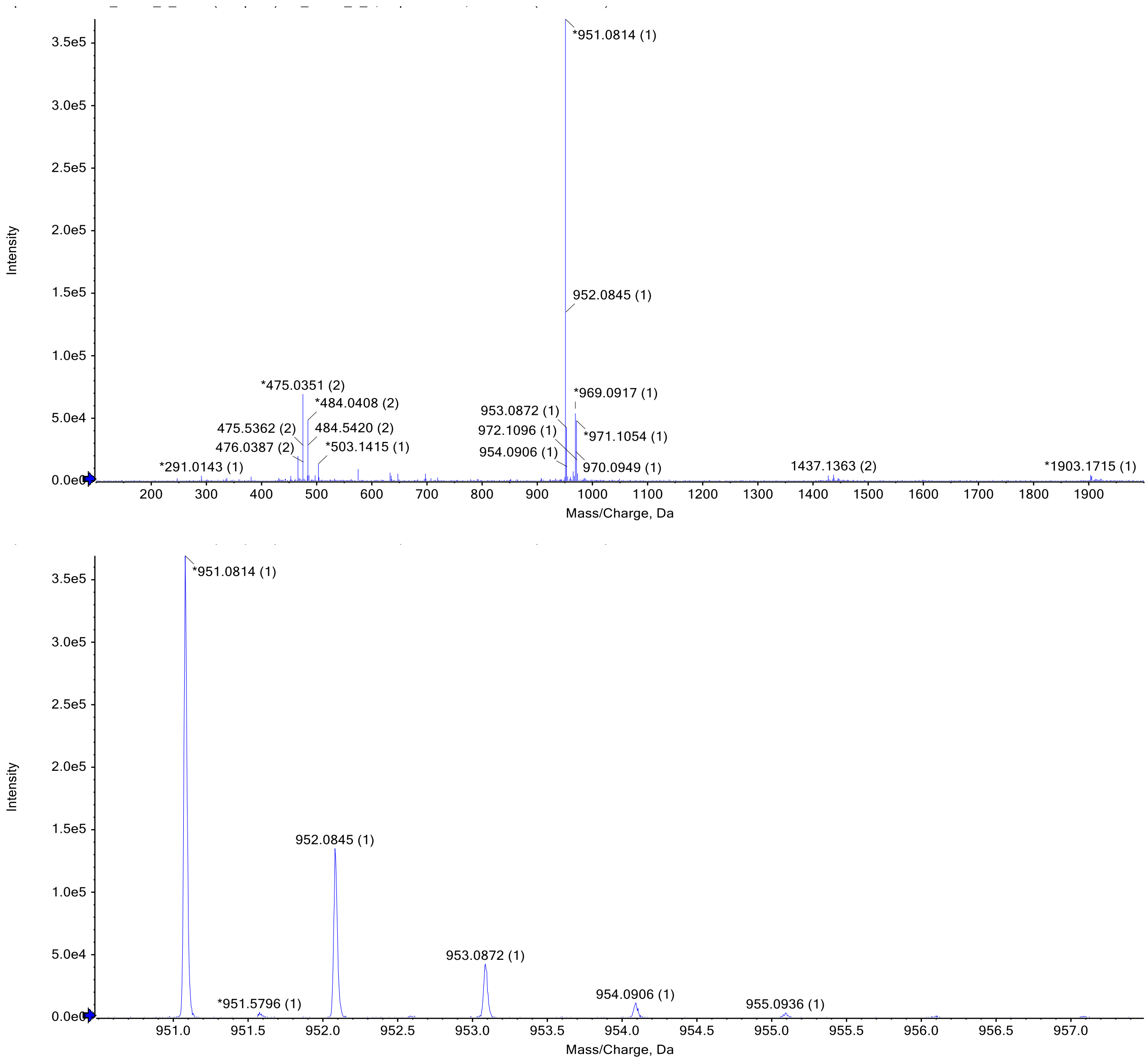

Figure S3. Negative HR-ESI/MS spectrum of geraniin.
